# Anti-tumour activity of tivozanib, a pan-inhibitor of VEGF receptors, in therapy-resistant ovarian carcinoma cells

**DOI:** 10.1038/srep45954

**Published:** 2017-04-06

**Authors:** Majid Momeny, Zahra Sabourinejad, Ghazaleh Zarrinrad, Farima Moghaddaskho, Haniyeh Eyvani, Hassan Yousefi, Shahab Mirshahvaladi, Ensieh M. Poursani, Farinaz Barghi, Arash Poursheikhani, Leila Dardaei, Davood Bashash, Mahmoud Ghazi-Khansari, Seyyed M. Tavangar, Ahmad R. Dehpour, Marjan Yaghmaie, Kamran Alimoghaddam, Ardeshir Ghavamzadeh, Seyed H. Ghaffari

**Affiliations:** 1 Haematology/Oncology and Stem Cell Transplantation Research Centre, Shariati Hospital, School of Medicine, Tehran University of Medical Sciences, Tehran, Iran; 2 Department of Pathology, Shariati Hospital, School of Medicine, Tehran University of Medical Sciences, Tehran, Iran; 3 Islamic Azad University, East Tehran Branch, Tehran, Iran; 4 Department of Medical Genetics, School of Medicine, Tehran University of Medical Sciences, Tehran, Iran; 5 Department of Molecular Systems Biology, Cell Science Research Centre, Royan Institute for Stem Cell Biology and Technology, Tehran, Iran; 6 Massachusetts General Hospital Cancer Centre, Charlestown, MA, USA; 7 Department of Haematology and Blood Banking, Faculty of Allied Medicine, Shahid Beheshti University of Medical Sciences, Tehran, Iran; 8 Department of Pharmacology, School of Medicine, Tehran University of Medical Sciences, Tehran, Iran; 9 Experimental Medicine Research Centre, Tehran University of Medical Sciences, Tehran, Iran

## Abstract

Epithelial ovarian cancer (EOC) is the most fatal gynaecological malignancy. Despite initial therapeutic response, the majority of advanced-stage patients relapse and succumb to chemoresistant disease. Overcoming drug resistance is the key to successful treatment of EOC. Members of vascular endothelial growth factor (VEGF) family are overexpressed in EOC and play key roles in its malignant progression though their contribution in development of the chemoresistant disease remains elusive. Here we show that expression of the VEGF family is higher in therapy-resistant EOC cells compared to sensitive ones. Overexpression of *VEGFR2* correlated with resistance to cisplatin and combination with VEGFR2-inhibitor apatinib synergistically increased cisplatin sensitivity. Tivozanib, a pan-inhibitor of VEGF receptors, reduced proliferation of the chemoresistant EOC cells through induction of G2/M cell cycle arrest and apoptotic cell death. Tivozanib decreased invasive potential of these cells, concomitant with reduction of intercellular adhesion molecule-1 (ICAM-1) and diminishing the enzymatic activity of urokinase-type plasminogen activator (uPA) and matrix metalloproteinase-2 (MMP-2). Moreover, tivozanib synergistically enhanced anti-tumour effects of EGFR-directed therapies including erlotinib. These findings suggest that the VEGF pathway has potential as a therapeutic target in therapy-resistant EOC and VEGFR blockade by tivozanib may yield stronger anti-tumour efficacy and circumvent resistance to EGFR-directed therapies.

Epithelial ovarian cancer (EOC) is the fifth most common cause of cancer death among women worldwide. It is estimated that approximately 22000 women are diagnosed with EOC in the United States and 14000 patients die from this disease each year^1^. Late-stage diagnosis, peritoneal metastasis and frequent development of chemoresistance restrain improvements in overall survival rate. First-line treatment for EOC includes debulking surgery followed by taxane/platinum-based regimens. Despite promising initial response, the majority of patients with advanced disease relapse and exhibit resistance to both chemotherapeutics and targeted therapies^2^.

Intrinsic and acquired resistance to chemotherapy are responsible for treatment failure in EOC^3^. Patients with the recurrent disease are treated with agents such as gemcitabine but clinical trials report that the median overall survival is still dismal^4^. There is, therefore, a pressing need to devise more efficacious treatments to overcome chemoresistance mechanisms and improve the outcome of EOC patients.

Angiogenesis, a multi-step process by which tumours develop new vasculature, is essential for tumour growth and metastasis^5^. The vascular endothelial growth factor (VEGF)/VEGF receptor (VEGFR) signalling pathway is the most promising angiogenic target due to its key roles in angiogenesis and tumour growth^6^,^7^. The VEGF family consists of seven ligands including VEGFA, VEGFB, VEGFC, VEGFD, VEGFE, placenta growth factor (PlGF) 1, and PlGF2. The tyrosine kinase receptors in this family include VEGFR type 1 (VEGFR1), VEGFR2 and VEGFR3^6^. Synthesized VEGF mimicking peptides have also been shown to bind to VEGF receptors, initiate VEGF-induced signalling and stimulate angiogenesis^8^.

Elevated expression of the VEGF ligands and receptors promotes malignant progression and correlates with poor prognosis in EOC^9^,^10^. High expression of VEGFA associates with advanced stage disease, development of malignant ascites and acquisition of an invasive phenotype^11^. Increased expression of VEGFC and VEGFR2 correlates with lymph node metastasis and peritoneal dissemination, a frequent cause of death in patients with primary advanced or recurrent EOC^12^,^13^. In this setting, blocking VEGFA activity in murine models of EOC halts tumour growth and ascites formation^14^. Altogether, these studies suggest that the VEGF family is importantly implicated in pathogenesis of EOC by influencing tumour growth and metastasis (via driving angiogenesis) and ascites formation (through stimulation of vascular permeability)^15^.

Evidence indicates that targeting angiogenesis is an effective therapeutic strategy in EOC and anti-angiogenic agents are among the most successful targeted therapies in this malignancy^16^,^17^. Patients treated with bevacizumab (anti-VEGFA mAb) alone or in combination with cytotoxic chemotherapies have demonstrated improvements in progression-free survival^18^,^19^. Addition of bevacizumab to several cytotoxic regimens improves response rate in patients with recurrent platinum-resistant disease^20^,^21^. While early clinical studies have determined remarkable activity of bevacizumab, lack of improvement in overall survival, considerable toxicity, frequent development of resistance, absence of a predictive biomarker and high cost of bevacizumab therapy highlight the need to establish novel and more efficacious anti-angiogenesis therapy in EOC^17^,^22^.

Tivozanib is a pan-VEGFR tyrosine kinase inhibitor that hampers angiogenesis and vascular permeability in tumour tissues^23^. Tivozanib has shown anti-tumour activities in xenograft models of colon, breast, lung, prostate, pancreas, glioblastoma and renal cell carcinoma^24^,^25^. In a phase I study in patients with advanced solid tumours, it has been found to be well tolerable with manageable side effects and durable clinical activity^26^. Tivozanib is currently under investigation in a phase II study in recurrent platinum-resistant ovarian cancer (NCT01853644)^27^. In the present study, we examined the mechanistic activity of tivozanib in therapy-resistant EOC cell lines.

## Results

### Chemosensitivity of the EOC cell lines

The sensitivity of a panel of EOC cell lines to certain chemotherapeutic agents and targeted therapies were determined by MTT assay and are summarized in Table 1. These data show that OVCAR3, SKOV3 and A2780CP cells exhibit multidrug-resistant behaviour. Moreover, A2780S and Caov4 cells show sensitivity to carboplatin, paclitaxel, doxorubicin, gemcitabine, erlotinib and cetuximab (Table 1, Supplementary Fig. 1).

### Expression of the VEGF family in the EOC cells

**Figure 1:**
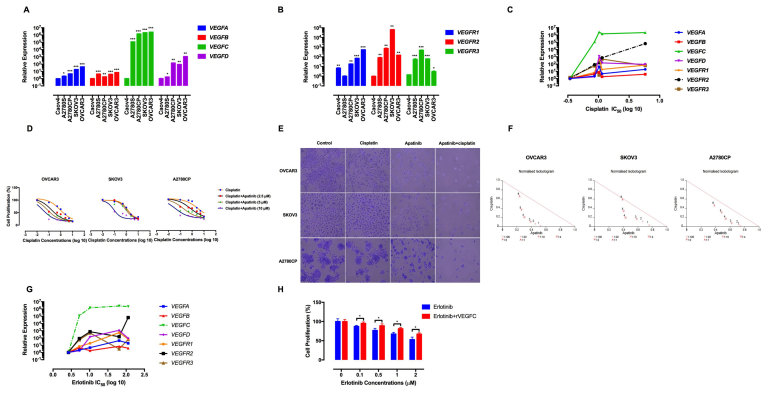
Expression of the VEGF family in the EOC cells. (**A,B**). Higher expression of *VEGFA, VEGFC, VEGFD, VEGFR1* and *VEGFR2* was observed in the multidrug-resistant OVCAR3, SKOV3 and A2780CP cells compared to the chemosensitive cell lines. Gene expression levels were normalized to *HPRT1* (**C**) Correlation of *VEGFR2* expression with resistance to cisplatin. EOC cell lines with higher expression of *VEGFR2* showed significantly higher cisplatin IC_50_ values. **(D)** The effect of the time-sequenced apatinib-cisplatin therapy on cell proliferation was investigated by MTT assay and shown by IC_50_ shift analysis. The cultures were pre-treated with apatinib (10 μM) followed by treatment with cisplatin (0.1, 0.5, 1, 2.5, 5 and 10 μg/mL) for 48 h. **(E)** The effect of apatinib-cisplatin therapy on cell viability was demonstrated by crystal violet staining. The cells were pre-treated with apatinib (10 μM) followed by treatment with 0.1 μg/mL of cisplatin for 48 h. The cultures were stained with crystal violet and imaged by an inverted microscope (images acquired at 10x magnification). **(F)** Normalised isobolograms of combination of apatinib and cisplatin. The data were analysed using the CalcuSyn software. The connecting line represents additivity. Data points located below the line indicate a synergistic drug-drug interaction and data points above the line indicate an antagonistic interaction. The numbers under the isobolograms indicate the doses of cisplatin and apatinib in combination. (**G**) Association of *VEGFC* expression and resistance to erlotinib. Higher expression of *VEGFC (r* = 0.93, *P* = 0.02) positively correlated with resistance to erlotinib. **(H)** The effect of exogenous VEGFC on proliferative response to erlotinib was determined by MTT assay. Caov4 cells were pre-treated with human recombinant VEGFC (rVEGFC) for 4 h, followed by treatment with erlotinib for 48 h. Data are given as mean ± SD. Statistically significant values of *p < 0.05, **p < 0.01, and ***p < 0.001 were determined compared with the control.

The expression of VEGF ligands and receptors in chemoresistant versus chemosensitive EOC cells is not yet examined. To explore potential association between chemoresponsiveness and expression of the VEGF family, their relative expression was investigated by qRT-PCR. This screening experiment revealed that the expression of *VEGFA, VEGFC, VEGFD, VEGFR1* and *VEGFR2* is higher in multidrug-resistant OVCAR3, SKOV3 and A2780CP cells compared to the chemosensitive ones (Fig. 1A,B).

The elevated expression of the VEGF family in the therapy-resistant EOC cells prompted us to examine possible correlation between their mRNA levels and chemoresponsiveness. We found that higher expression of *VEGFR2* associates with resistance to cisplatin by Pearson’s correlation (Fig. 1C). The correlation coefficient (*r*) between the *VEGFR2* expression and cisplatin concentrations was 0.8876 (*P* = 0.0404). To further confirm that VEGFR2 may contribute to cisplatin resistance, we determined the effects of apatinib, a VEGFR2 specific inhibitor, on proliferative response of the chemoresistant EOC cells to cisplatin. For the combination therapy, we followed a time-staggered treatment protocol as described by Lee *et al*.^28^. The cells were pre-treated with apatinib for 4 h, followed by treatment with cisplatin for 48 h. Combination with apatinib synergistically increased sensitivity to cisplatin (Fig. 1D–F, Supplementary Table 1).

We also found a positive correlation between erlotinib resistance and higher expression of *VEGFC* (Fig. 1G). Moreover, pre-treatment with human recombinant VEGFC (10 ng/mL) decreased anti-proliferative effects of erlotinib in the chemosensitive Caov4 cells (Fig. 1H). Altogether, these data suggest that VEGFR2 and VEGFC might contribute to resistance to cisplatin and erlotinib, respectively.

### Tivozanib inhibits proliferation, clonogenic potential and anoikis resistance

**Figure 2:**
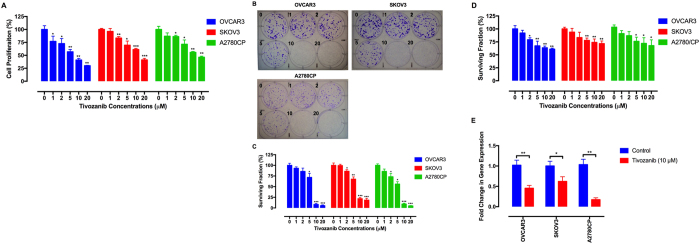
Tivozanib inhibits proliferation, clonal growth and anoikis resistance. (**A**) The effect of tivozanib on cell viability was estimated by MTT assay. (**B,C**) Clonogenic assay was conducted to evaluate the effect of tivozanib on clonal proliferation. (**D**) Anoikis resistance assay was performed with cell culture on poly-HEMA–coated culture dishes for 48 h and the proportion of viable cells was measured by MTT assay. **(E)** qRT-PCR was carried out to determine whether tivozanib-mediated decrease in the surviving fraction is due to down-regulation of the anoikis resistance marker *BCL2*. Gene expression levels were normalized to *HPRT1*. Data are given as mean ± SD, normalized to the vehicle-treated control group. Statistically significant values of *p < 0.05, **p < 0.01, and ***p < 0.001 were determined compared with the control.

MTT assay was carried out to determine the effect of tivozanib on proliferation of the therapy-resistant EOC cells. Treatment of these cells with tivozanib reduced their proliferation (Fig. 2A). Moreover, tivozanib diminished their clonogenic growth (Fig. 2B,C). In immortalized cells, detachment from the extracellular matrix induces anoikis, a special type of apoptosis^29^. Acquisition of resistance to anoikis is a prerequisite for EOC cells to survive in the ascites before forming metastatic foci^30^. Using an anoikis resistance assay, we found that tivozanib reduced surviving fraction of the EOC cells (Fig. 2D). Tivozanib decreased the expression of the anoikis resistance marker *BCL2*^31^, suggesting that the diminished surviving fraction by tivozanib is due to enhanced anoikis (Fig. 2E).

### Tivozanib induces G2/M cell cycle arrest and apoptosis

**Figure 3:**
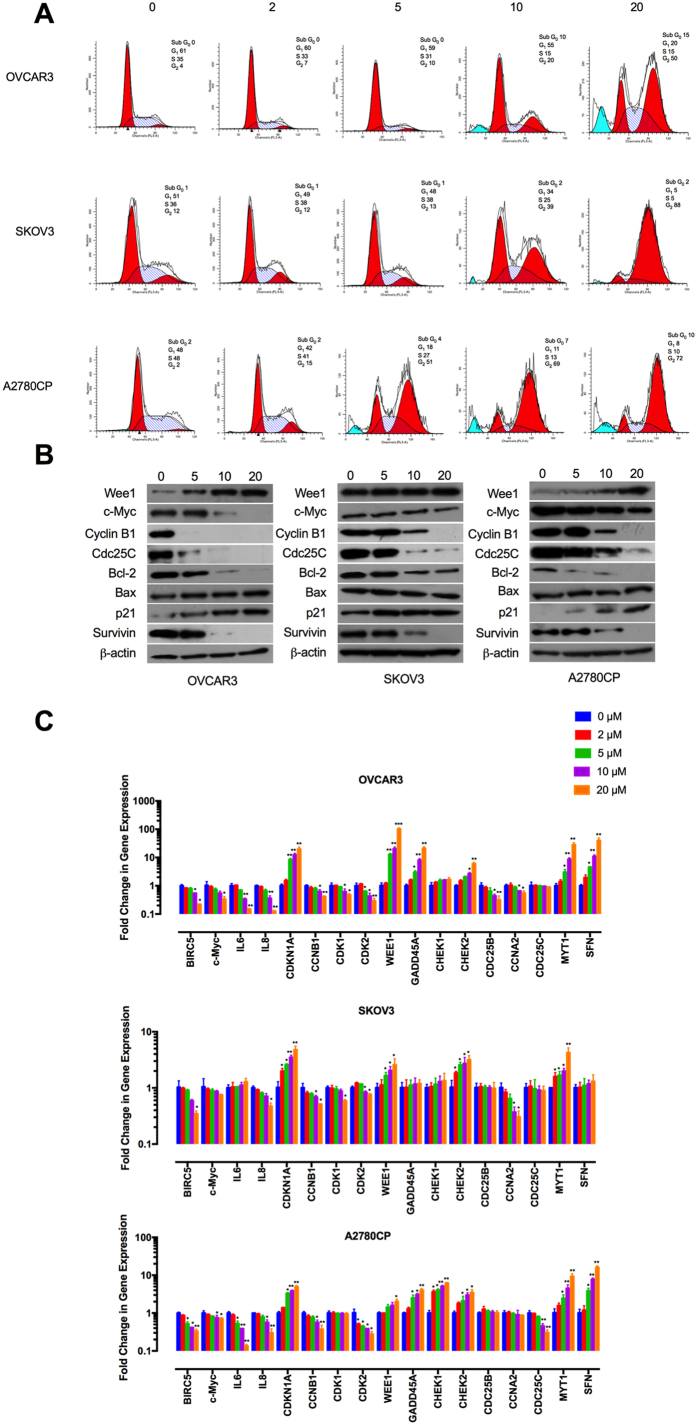
Tivozanib induces G2/M cell cycle arrest and apoptosis. (**A**) Following treatment with tivozanib for 48 h, the cell pellets were fixed and incubated with propidium iodide to analyse the cell cycle distribution on a flow cytometer. The concentrations of tivozanib were 2, 5, 10 and 20 μM. The graphs are representative of three independent experiments with similar results. (**B**) Protein lysates from tivozanib-treated cells were subjected to Western blotting and probed with the indicated antibodies. β-actin was used as the loading control. The concentrations of tivozanib were 5, 10 and 20 μM. The blots are representative of three independent experiments with similar outcomes. (**C**) The cells were treated with tivozanib for 48 h then total RNA was harvested for qRT-PCR analysis. Gene expression levels were normalized to *HPRT1*. Data are given as mean ± SD. Statistically significant values of *p < 0.05, **p < 0.01, and ***p < 0.001 were determined compared with the control. *IL*, interleukin; *CDKN1A*, cyclin-dependent kinase inhibitor 1 A; *CCNB1*, cyclin B1; *CDK*, cyclin-dependent kinase; *GADD45A*, growth arrest and DNA damage inducible alpha; *CHEK*, checkpoint kinase; *CDC25*, cell division cycle 25; *CCNA2*, cyclin A2; *MYT1*, myelin transcription factor 1; *SFN*, stratifin.

Due to the anti-proliferative effects of tivozanib, we asked if tivozanib inhibits cell cycle progression or affects apoptosis. In OVCAR3 and A2780CP cells, tivozanib increased the percentage of cells in G2/M phase while decreasing the G1/S fraction. Moreover, a small number of cells underwent apoptotic cell death, as indicated by appearance of a sub- G0/G1 population. SKOV3 cells treated with tivozanib displayed an increase in the G2/M population (Fig. 3A).

We next determined the effects of tivozanib on expression of genes and proteins that regulate the G2/M transition. Activation of Cdc2/cyclin B kinase complex is a pivotal step in mitotic initiation. Wee1 and myelin transcription factor 1 (encoded by *MYT1*) are cell cycle-regulated kinases that block mitotic entry via phosphorylation of Cdc2 while Cdc25C dephosphorylates Cdc2 and permits entry into mitosis^32^,^33^. Checkpoint kinase 2 (encoded by *CHEK2*) is a putative tumour suppressor that prevents mitotic progression by inhibiting Cdc25C^34^.

Tivozanib decreased both Cdc25C and cyclin B1 protein expression (Fig. 3B, Supplementary Fig. 2). Both mRNA and protein levels of the G2/M checkpoint regulator p21 (encoded by *CDKN1A*) were increased following tivozanib treatment (Fig. 3B,C, Supplementary Fig. 2). Moreover, tivozanib increased the mRNA levels of *WEE1, MYT1* and *CHEK2* (Fig. 3C). These data show that tivozanib inhibits proliferation of the EOC cells through a G2/M cell cycle arrest and induction of apoptosis.

### Tivozanib reduces invasive abilities of the EOC cells

**Figure 4:**
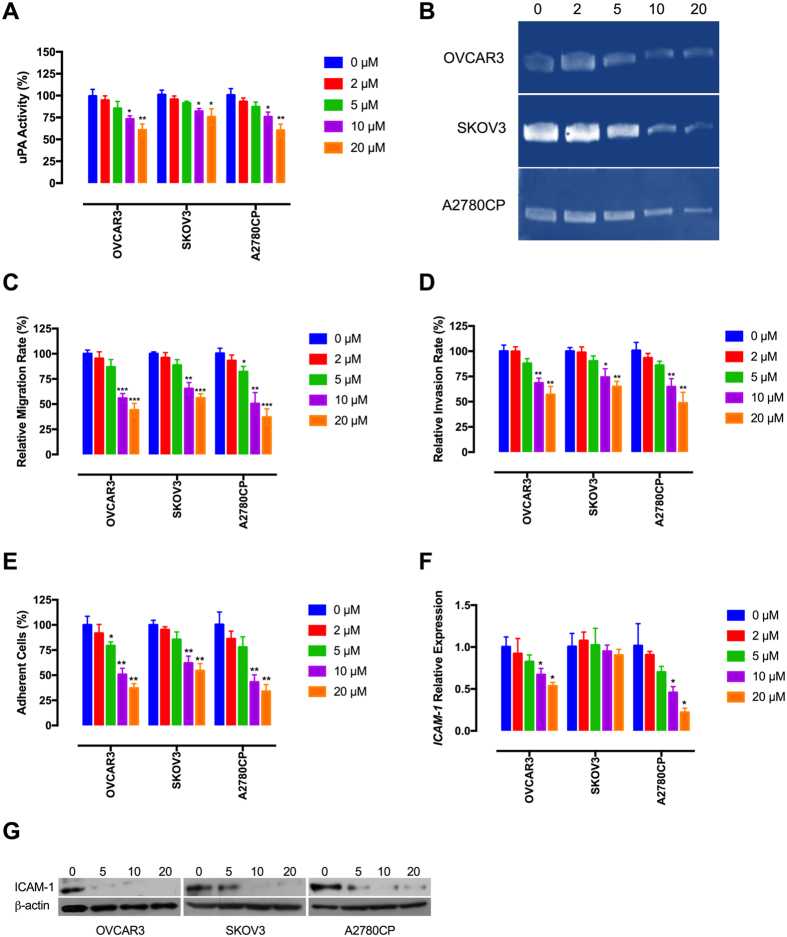
(**A**) Tivozanib inhibits uPA activity. The cells were treated with tivozanib for 48 h and equal amounts of total secreted protein from each sample were subjected to a chromogenic substrate, which is cleaved by active uPA and produces a colorimetrically detectable product. (**B**) Tivozanib inhibits enzymatic levels of MMP-2. The conditioned media from tivozanib-treated cells was separated on a non-reducing polyacrylamide gel containing gelatin A. Gelatinolytic activities are visualized as clear bands against the blue background of stained gelatin. The concentrations of tivozanib were 2, 5, 10 and 20 μM. The zymograms are representative of three independent experiments with similar results. The gels were cropped and the full-length gels are presented in Supplementary Fig. 3 (**C,D**) Tivozanib hinders migration and invasion. The cells were placed into 8-μm porous culture inserts, treated with tivozanib and allowed to migrate for 48 h. The migrated cells on the lower surface of the inserts were quantified by crystal violet staining. For invasion assay, the cells were placed into matrigel-coated inserts and allowed to invade through the matrigel layer for 48 h. (**E**) Tivozanib decreases adhesive potential of the EOC cells. Tivozanib-treated cells were seeded into collagen I-coated culture dishes then the adhesive cells were stained, lysed and the optical densitometry was read. **(F)** The effect of tivozanib on *ICAM-1* expression was measured by qRT-PCR. **(G)** The cells were treated with tivozanib then protein lysates were subjected to Western blotting and probed with ICAM-1 antibody. β-actin was used as the loading control. The concentrations of tivozanib were 5, 10 and 20 μM. The blots are representative of three independent experiments with similar results. Data are given as mean ± SD. Statistically significant values of *p < 0.05, **p < 0.01, and ***p < 0.001 were determined compared with the control.

Interplays between the VEGF pathway and extracellular proteinases including urokinase plasminogen activator (uPA) and matrix metalloproteinases (MMPs) are implicated in peritoneal spread of EOC cells^35^. Evidence indicates that the VEGF/VEGFR loop drives EOC invasion through induction of uPA and MMP-2^36^,^37^. Conversely, MMP-2 contributes to release of biologically active VEGFA and ascites formation^38^. Our findings show that tivozanib reduced enzymatic levels of uPA and MMP-2 (Fig. 4A,B, Supplementary Fig. 3).

The VEGF/VEGFR pathway has been shown to promote metastatic dissemination of EOC cells^39^,^40^. We next determined if tivozanib-mediated inhibition of uPA and MMP-2 associates with attenuation of migration and invasion in the EOC cells. Using Transwell assays, the resulting data demonstrate that tivozanib diminished migratory and invasive behaviour of these cells (Fig. 4C,D).

A major route for the metastatic spread of EOC cells is by attachment to the mesothelium lining in the peritoneal cavity, a critical step to establish foci and penetrate the underlying stroma^41^. To investigate whether tivozanib attenuates adhesive properties of the EOC cells, we assayed adhesion of tivozanib-treated cells to collagen I which is a substrate for a range of cell adhesion molecules. Tivozanib reduced adhesion of these cells to collagen I, concomitant with suppression of intercellular adhesion molecule-1 (ICAM-1) (Fig. 4E–G), a cell surface adhesion molecule that mediates tumour cell binding to the mesothelium and enhances tumour invasion^42^,^43^.

### Effects of tivozanib on VEGFR2, AKT, ERK1/2 and NF-κB pathways

**Figure 5:**
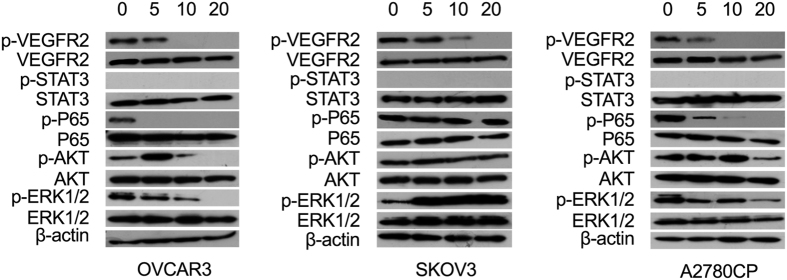
Effects of tivozanib on activation of pertinent oncogenic pathways. The cells were treated with tivozanib for 48 h then whole cell lysates were prepared and resolved by SDS PAGE. Samples were blotted for the phospho-form and re-probed for the respective total form of VEGFR2, STAT3, NF-κB p65, AKT and ERK1/2. β-actin was used as the loading control. The concentrations of tivozanib were 5, 10 and 20 μM. The blots are representative of three independent experiments with similar results.

The VEGF/VEGFR pathway causes a cascade of downstream events including activation of Ras/MEK/ERK axis^44^. In ovarian carcinoma tissues, activation of STAT3 correlates with expression of VEGFA, VEGFR1 and VEGFR2^45^. In addition, a positive association between expression of VEGFR2 and AKT activation has been demonstrated in EOC clinical samples^46^. Western blot analysis was applied to explore the effects of tivozanib-mediated inhibition of p-VEGFR2 on AKT, ERK1/2, STAT3 and NF-κB pathways. Tivozanib at higher doses attenuated AKT, ERK1/2 and NF-κB pathways in OVCAR3 and A2780CP cells. In comparison, tivozanib treatment induced p-ERK1/2 in SKOV3 cells, suggesting that ERK1/2 activation might be a compensatory mechanism for inhibition of VEGFR signalling in these cells (Fig. 5).

### Synergistic activity of tivozanib with EGFR-targeted therapies

**Figure 6:**
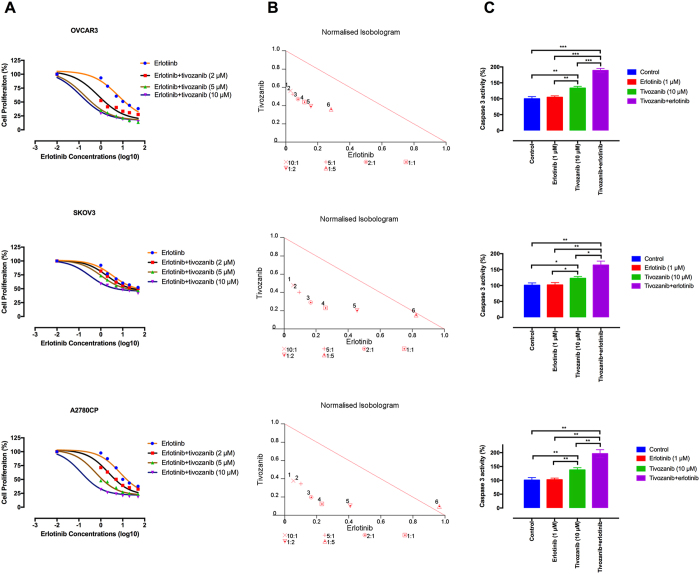
Combined treatment with tivozanib and erlotinib results in anti-proliferative and pro-apoptotic synergism. (**A**) The effect of the time-sequenced tivozanib-erlotinib therapy on cell proliferation was investigated by MTT assay and shown by IC_50_ shift analysis. **(B)** Normalised isobolograms of combination of tivozanib (10 μM) and erlotinib (1, 2, 5, 10, 20 and 50 μM). The data were analysed using the CalcuSyn software. The connecting line represents additivity. Data points located below the line indicate a synergistic drug-drug interaction and data points above the line indicate an antagonistic drug-drug interaction. The numbers under the isobolograms indicate the doses of tivozanib and erlotiinb in combination **(C)** The effect of combined tivozanib-erlotinib treatment on activation of caspase 3 was measured by a colorimetric caspase 3 activity assay. Data are given as mean ± SD. Statistically significant values of *p < 0.05, **p < 0.01, and ***p < 0.001 were determined compared with the control.

EGFR overexpression occurs in 35–70% of EOC patients^47^. Preclinical data suggest that resistance to anti-EGFR targeted therapies results from increased tumour angiogenesis^48^. We therefore asked if VEGFR blockade by tivozanib increases sensitivity to EGFR-directed therapies. For the combination therapy, the cells were pre-treated with tivozanib for 4 h, followed by treatment with anti-EGFR therapies including erlotinib, gefitinib and cetuximab for 48 h. The time-sequenced tivozanib-anti-EGFR therapy had a synergistic effect on growth inhibition and activation of caspase-3, an indicator of apoptosis (Fig. 6, Table 2, Supplementary Figs 4 and 5 and Supplementary Tables 3 and 4). These data suggest that VEGFR blockade by tivozanib enhances sensitivity to the EGFR-directed therapies in the EOC cells.

## Discussion

The VEGF family is aberrantly expressed in EOC^9^,^49^. High levels of VEGFA correlate with advanced tumour stage, ascites formation, distant metastases and poor overall survival^50^,^51^. Spannuth *et al* have demonstrated higher expression of VEGFR2 in malignant ovarian samples compared to benign or borderline tumours, which showed a correlation with tumour grade and aggressiveness^39^,^52^. Moreover, VEGFA suppresses immune-mediated anti-tumour responses in EOC^53^. Although these findings indicate that the VEGF/VEGFR loop promotes malignant progression in EOC, the role of this pathway in driving the chemoresistant disease is largely unknown.

Emerging data indicate that the VEGF ligands act as survival factors for tumour cells that express the receptors^54^,^55^,^56^. In colorectal cancer cells, *VEGFA* depletion reduces cell survival and enhances chemosensitivity via blockade of AKT and ERK1/2 pathways^57^. An autocrine VEGFA/VEGFR2 pathway opposes apoptosis in leukaemia cells through induction of the anti-apoptotic protein Bcl-2^58^. An angiogenic loop of VEGFC/VEGFR3 protects leukemic cells from pro-apoptotic effects of chemotherapy^59^ and blockade of VEGFR3 induces chemosensitisation in EOC cells^60^. Consistent with this, our data demonstrate that high expression of *VEGFR2* and *VEGFC* correlates with resistance to cisplatin and erlotinib, respectively. Collectively, these findings suggest that the VEGF family plays important roles in therapy resistance and blocking all the VEGF receptors may provide advantages over single-targeted therapies.

Abnormalities in cell cycle mediators such as cyclins, cyclin-dependent kinases (CDKs) and their inhibitors are thought to be early events in the pathogenesis of EOC and provide an unchecked growth advantage^61^. Deregulation of the G2/M regulatory proteins p21, cyclin B1 and Cdc25C correlates with poor survival in EOC^62^. Exogenous expression of p21, depletion of *CCNB1* and shRNA suppression of *CDC25C* inhibit growth and induce apoptosis^63^,^64^,^65^,^66^. These findings suggest that the cell cycle regulatory network is a promising therapeutic target to halt growth and proliferation of EOC cells^67^,^68^. In line with this, the results of the present study show that VEGFR blockade by tivozanib retards proliferation of the chemoresistant EOC cells through a G2/M cell cycle arrest via up-regulation of p21 and down-modulation of cyclin B1 and Cdc25C.

EOC is a highly metastatic malignancy characterized by peritoneal dissemination. An essential step in the peritoneal spread of EOC is adhesion and implantation of the tumour cells to the mesothelial cells lining the peritoneum^69^. Cell adhesion molecules including ICAM-1 mediate the adhesion process^42^. Moreover, extracellular proteinases MMP-2 and uPA play central roles in mesothelial invasion and their blocking reduces peritoneal metastasis^70^,^71^,^72^. Evidence indicates that increasing EOC migration and invasion is a mechanism through which the VEGF family promotes peritoneal dissemination^73^. The findings of this study reveal that tivozanib reduces adhesive and invasive characteristics of the EOC cells through inhibition of ICAM-1, uPA and MMP-2 and suggest that it might have clinical applications in translational oncology to pre-empt peritoneal dissemination.

Elevated expression of VEGFA drives resistance to anti-EGFR therapies^74^. Acquired resistance of xenograft models of squamous cell carcinoma to anti-EGFR monoclonal antibodies associates with enhanced levels of VEGFA^48^. Moreover, increased expression of VEGFA contributes to development of gefitinib-resistant colon cancer cells, which is abrogated by treatment with a VEGFR2 inhibitor^75^. In the present study, we found that erlotinib-resistant EOC cells exhibit over-expression of *VEGFC* and pre-treatment with tivozanib synergistically enhanced erlotinib anti-proliferative activity. Altogether, these data suggest that VEGFR blockade by tivozanib induce sensitisation to the EGFR-directed therapies in the EOC cells.

Members of the Bcl-2 family of proteins play an essential role in chemosensitivity in EOC^76^,^77^. High levels of Bax correlate with increased response to paclitaxel chemotherapy and reduced risk of relapse^78^. ABT737, a Bcl-2 family inhibitor, sensitises EOC cells to carboplatin and co-delivery of survivin shRNA and paclitaxel synergistically induces apoptosis^79^,^80^. Our findings indicate that tivozanib-induced sensitisation to the anti-EGFR therapies might be through down-regulation of the anti-apoptotic proteins survivin (encoded by *BIRC5*) and Bcl-2.

Taken together, our data suggest that the VEGF/VEGFR loop may have potential as a therapeutic target in the chemoresistant EOC and provide new insight into the mechanistic activities of tivozanib. Blockade of VEGF receptors by tivozanib reduced proliferative and invasive characteristics of the drug-resistant EOC cells. Combination of tivozanib with the EGFR-directed therapies displayed synergistic activity on cell growth inhibition and induction of apoptosis, suggesting that anti-VEGFR-targeted approaches induce sensitisation to the EGFR-directed therapies. Further *in vivo* studies are warranted to explore the anti-tumour activity of tivozanib alone or in combination with EGFR inhibitors in chemoresistant EOC.

## Materials and methods

### Antibodies and chemicals

Antibodies were obtained as follows: AKT, p-AKT (Ser473; clone D9E), ERK1/2 and p-ERK1/2 (Thr202/Tyr204; clone 197G2), p-VEGFR2 (Tyr1175; clone 19A10), p-NF-κB p65 (Ser536; clone 93H1) (Cell Signalling Technology); VEGFR2 (clone A-3), STAT3 (clone C-20), p-STAT3 (clone B-7), NF-κB p65 (clone C-20), ICAM-1 (clone H-108), Bcl-2 (clone N-19), Bax (clone N-20), survivin (clone FL-142), p21 (clone C-19), c-Myc (clone 9E10), cyclin B1 (clone GNS1), Wee1 (clone C-20), Cdc25C (clone C-20) and β-actin (Santa Cruz Biotechnology).

Tivozanib and apatinib (a highly selective VEGFR-2 tyrosine kinase inhibitor) were purchased from AdooQ BioScience (Irvine, CA, USA) and were dissolved in DMSO. The final concentrations of DMSO did not exceed than 0.1% [v/v] in all the treatments. Erlotinib and gefitinib (EGFR small-molecule tyrosine kinase inhibitors) were obtained from ChemieTek (Indianapolis, IN, USA). Cetuximab (a ligand-blocking anti-EGFR mAb), cisplatin and doxorubicin (DNA-damaging drugs), paclitaxel (a taxane inhibitor of microtubule disassembly), vincristine (a mitosis-blocking agent), carboplatin (an alkylating agent) and gemcitabine (a nucleoside analogue which inhibits DNA synthesis) were purchased from the pharmacy of Shariati hospital (Tehran, Iran). Poly-hydroxyethylmethacrylate polymer (poly-HEMA) was obtained from Santa Cruz Biotechnology. Human recombinant VEGFC (rVEGFC) was purchased from Peprotech.

### Human ovarian carcinoma cell lines

Human ovarian carcinoma cell lines were obtained from National Cell Bank of Iran (NCBI; Tehran, Iran). These include A2780CP (adenocarcinoma), A2780S (adenocarcinoma), Caov4 (originated from metastatic fallopian tube mass), OVCAR3 (originated from ovarian cancer ascites) and SKOV3 (originated from ovarian cancer ascites)^81^. All the cell lines were authenticated by STR profiling using Cell ID^TM^ system (Promega) and were routinely checked for mycoplasma infection. Cell cultures were maintained at 37 °C in 5% CO_2_ in a humidified incubator and cultured according to the NCBI recommendations.

### Cytotoxicity assays

The EOC cells in logarithmic growth phase were plated (2 × 10^3^ cells/well) in 96-well plates. After incubation at 37 °C for 24 h, the cultures were exposed to desired concentrations of the chemotherapeutics for 48 h and the proportion of viable cells was determined by MTT assay. Vehicle-treated cells were used as the control group. Cytotoxicity was shown as IC_50_ values calculated from full dose–response curves. Synergism was determined by calculation of the combination index (CI) according to Chou and Talalay^82^ using the CalcuSyn software (Biosoft, Cambridge, UK). CI < 1, CI = 1, and CI > 1 represent synergism, additive effects, and antagonism of the 2 drugs, respectively.

### Crystal violet staining

The cells were plated at a density of 6 × 10^4^ cells in 6-well plates and treated with the drugs for 48 h. The cultures were then washed with PBS, fixed with ice-cold methanol and stained with crystal violet (0.5% w/v). The images were acquired with an inverted microscope.

### Colony formation assay

Cells were seeded into 6-well plates with a density of 200–400 cell/well. After 12 h, the cells were treated with increasing concentrations of tivozanib for 48 h. The media was changed to drug-free media and the cells were incubated at 37 °C in 5% CO_2_ for 10 d. The cultures were fixed in ice-cold methanol for 20 min at room temperature and stained with crystal violet solution (0.5% w/v). The colonies were counted by naked eyes and the surviving fraction (SF) was estimated as: (mean colony counts)/(cells plated) × (plating efficiency), where plating efficiency (PE) was determined as (mean colony counts)/(cells plated for controls).

### Anoikis resistance assay

Poly-HEMA was solubilized in 95% ethanol (20 mg/mL) and then 25 μL of this solution was placed in 96-well plates and dried in a tissue culture hood. Anoikis was induced by culturing 5 × 10^3^ cells on poly-HEMA coated plates in the medium containing increasing concentrations of tivozanib. The cell suspension cultures were maintained in a humidified 5% CO_2_ incubator at 37 °C for 48 h. One hundred μL of MTT solution (0.5 mg/mL) was added to each well and the cells were further incubated at 37 °C for 2 h. The precipitated formazan was dissolved in DMSO and the optical densitometry was measured at 570 nm.

### Analysis of gene expression by quantitative reverse transcription-PCR

The quantitative reverse transcription-PCR (qRT-PCR) analysis was performed on a StepOne Plus instrument (Applied Biosystems) using RealQ-PCR Master Mix kit (Ampliqon, Copenhagen, Denmark). Thermal cycling conditions involved an activation step for 15 min at 95 °C followed by 40 cycles including a denaturation step for 15 s at 95 °C and a combined annealing/extension step for 1 min at 60 °C. The primers used are listed in Supplementary Table 2. The target gene expression levels were normalized to hypoxanthine phosphoribosyl transferase1 (*HPRT1*) levels in the same reaction. For calculations, 2 ^–ΔΔC^_T_ formula was used, with ΔΔC_T_ = (C_T *Target*_ − C_T *HPRT1*_) experimental sample − (C_T *Target*_ – C_T *HPRT1*_) control samples, where C_T_ is cycle threshold.

### Western blot analysis

Total protein extracts were prepared in RIPA buffer (50 mM Tris-HCl, pH 8.0, 150 mM NaCl, 1.0% NP-40, 0.5% sodium deoxycholate and 0.1% SDS) containing protease and phosphatase inhibitors (Roche Molecular Biochemicals) for 30 min at 4 °C. Fifty to hundred μg of lysate was resolved by SDS-PAGE, transferred to PVDF membrane (Membrane Solutions, TX, USA) then probed with primary and horseradish peroxidase (HRP)-conjugated secondary antibodies (Sigma). β-actin was used as the loading control and proteins were detected using a BM chemiluminescence detection kit (Roche Molecular Biochemicals).

### Cell cycle analysis

Propidium iodide staining was conducted for detection of DNA content. Following tivozanib treatment for 48 h, harvested cells were washed in ice-cold PBS, fixed in 70% ethanol and stored at −20 °C overnight. Vehicle-treated cells were used as the control group. The cell pellets were then incubated with RNase A (100 μg/mL) (Sigma), propidium iodide (50 μg/mL) (Sigma) and 0.05% Triton X-100. Cellular DNA content was analyzed on a FACSCalibur (BD Bioscience) flow cytometer equipped with CellQuest Pro software.

### Zymography

Equal amounts of secreted protein from the conditioned media of tivozanib-treated and vehicle-treated cells were applied to 10% polyacrylamide gels copolymerized with 1 mg/mL gelatin A (Sigma). After electrophoresis, gels were rinsed in 2.5% Triton X-100 (2 × 15 min) to remove SDS, followed by incubation at 37 °C overnight in incubation buffer (0.15 M NaCl, 10 mM CaCl_2_, 0.02% NaN_3_ in 50 mM Tris-HCl, pH 7.5). The gels were then stained (0.5% Coomassie Brilliant Blue) and destained with 7% methanol and 5% acetic acid. Areas of enzymatic activity appeared as clear bands over the dark background.

### Urokinase-type plasminogen activator activity assay

Urokinase-type plasminogen activator (uPA) activity was assayed with a uPA-specific chromogenic substrate according to the manufacturer’s instructions (Millipore). Equal amounts of protein from the uPA-containing conditioned media were added to the chromogenic substrate and incubated at 37 °C for 1 h. The samples were then read at 405 nm.

### Cell adhesion

After treatment with tivozanib for 48 h, the cells were counted and equal cell number from both tivozanib-treated and vehicle-treated groups was seeded in collagen I-coated 60 mm dishes (Biocoat Cell Environments; Becton Dickinson). Following incubation for 15 min at 37 °C, the cells were washed twice with cold PBS, stained with 0.5% crystal violet, lysed with 30% acetic acid and the optical densitometry was measured at 590 nm.

### Transwell cell migration and invasion

Cell migration and invasion assays were carried out as described earlier^83^.

### Caspase 3 activity assay

To assess induction of apoptosis, a colorimetric caspase 3 activity assay was employed according to the manufacturer’s protocol (Sigma). Briefly, cell lysates from both adherent and floating cells were centrifuged at 20000 × g for 10 min. Twenty μg of the supernatant was incubated with 85 μL of assay buffer plus 10 μL of caspase 3 substrate acetyl-Asp- Glu-Val-Asp p-nitroanilide (Ac-DEVD-pNA) in a 96-well plate at 37 °C for 12 h. The samples were then read at 405 nm in an ELISA reader.

### Statistical analysis

All data were evaluated in triplicate against vehicle-treated control cells and collected from three independent experiments. Data were graphed and analysed using GraphPad Prism Software 6.0 using one-way ANOVA and the unpaired two-tailed Student’s *t* test. All data are presented as mean ± standard deviation (SD).

## Additional Information

**How to cite this article**: Momeny, M. *et al*. Anti-tumour activity of tivozanib, a pan-inhibitor of VEGF receptors, in therapy-resistant ovarian carcinoma cells. *Sci. Rep.* **7**, 45954; doi: 10.1038/srep45954 (2017).

**Publisher's note:** Springer Nature remains neutral with regard to jurisdictional claims in published maps and institutional affiliations.

## Supplementary Material

Supplementary Information

## Figures and Tables

**Table 1:** Chemosensitivities of a panel of EOC cell lines to certain chemotherapeutics and targeted therapies.

Chemosensitivity (IC_50_)^a^								
Cell Lines	Cisplatin (μg/mL)	Carboplatin (μg/mL)	Paclitaxel (μg/mL)	Doxorubicin (ng/mL)	Vincristine (ng/mL)	Gemcitabine (ng/mL)	Erlotinib (μM)	Cetuximab (μg/mL)
**SKOV3**	5.8	71	5.4	696	>1000	24	114	>100
**A2780CP**	1.145	51	1.4	600	37	27	10.3	>100
**A2780S**	0.8634	4.6	0.2	4.1	32	16	5.2	82
**Caov4**	0.3	2.7	0.1	5.1	3.4	4.6	2.6	44

^a^Chemosensitivity is expressed as IC_50_ for each cell line, which is the concentration of drug that caused a 50% reduction in proliferation compared to vehicle-treated cells.

**Table 2:** Combination index (CI) and dose reduction index (DRI) of tivozanib and erlotinib combination in OVCAR3, SKOV3 and A2780CP cells.

Concentrations (μM)		fa	CI	DRI	
Tivozanib	Erlotinib			Tivozanib	Erlotinib
**OVCAR3**					
10	1	0.65	0.59	1.8	35.2
10	2	0.69	0.57	1.9	22.5
10	5	0.75	0.55	2.1	13.2
10	10	0.79	0.56	2.3	8.5
10	20	0.83	0.55	2.5	6.4
10	50	0.86	0.64	2.8	3.5
**SKOV3**					
10	1	0.41	0.54	2.1	17.3
10	2	0.44	0.5	2.5	10.5
10	5	0.49	0.46	3.4	6
10	10	0.52	0.49	4.3	3.9
10	20	0.54	0.66	4.9	2.2
10	50	0.58	1	6.5	1.2
**A2780CP**					
10	1	0.52	0.43	2.6	18.6
10	2	0.54	0.44	2.9	10
10	5	0.65	0.36	5	6.1
10	10	0.73	0.36	8	4.3
10	20	0.75	0.51	9.4	2.5
10	50	0.76	1.06	10.1	1

DRI represents the order of magnitude of dose reduction that is allowed in combination for a given degree of effect as compared with the dose of each drug alone. “fa” denotes fraction affected.
