# Supplementary material for: Anti-tumour activity of tivozanib, a pan-inhibitor of VEGF receptors, in therapy-resistant ovarian carcinoma cells
Source: Sci Rep. 2017 Apr 6;7:45954. doi: 10.1038/srep45954 (PMC5382685; doi:10.1038/srep45954)
Supplement: Supplementary Information [file srep45954-s1.pdf]

Anti-tumour activity of tivozanib, a pan-inhibitor of VEGF receptors, in therapy-resistant ovarian carcinoma cells

Majid Momeny[1,+], Zahra Sabourinejad[2, 3, +], Ghazaleh Zarrinrad[1,+], Farima Moghaddaskho[1], Haniyeh Eyvani[1], Hassan Yousefi[4], Shahab Mirshahvaladi[5], Ensieh M. Poursani[1], Farinaz Barghi[1], Arash Poursheikhani[4], Leila Dardaei[6], Davood Bashash[7], Mahmoud Ghazi-Khansari[8], Seyyed M. Tavangar[2], Ahmad R. Dehpour[8], Marjan Yaghmaie[1], Kamran Alimoghaddam[1], Ardeshir Ghavamzadeh[1], Seyed H. Ghaffari[1,*]

[1]Haematology/Oncology and Stem Cell Transplantation Research Centre, Shariati Hospital, School of Medicine, Tehran University of Medical Sciences, Tehran, Iran
[2]Deparmtent of Pathology, Shariati Hospital, School of Medicine, Tehran University of Medical Sciences, Tehran, Iran
[3]Islamic Azad University, East Tehran Branch, Tehran, Iran
[4]Deparmtent of Medical Genetics, School of Medicine, Tehran University of Medical Sciences, Tehran, Iran
[5]Department of Molecular Systems Biology, Cell Science Research Centre, Royan Institute for Stem Cell Biology and Technology, Tehran, Iran
[6]Massachusetts General Hospital Cancer Centre, Charlestown, MA, USA
[7]Department of Haematology and Blood Banking, Faculty of Allied Medicine, Shahid Beheshti University of Medical Sciences, Tehran, Iran
[8]Department of Pharmacology, School of Medicine, Tehran University of Medical Sciences, Tehran, Iran

[+]These authors contributed equally to this work

*Correspondence to: Seyed H. Ghaffari, email: shghaffari200@yahoo.com

Keywords: Epithelial ovarian cancer, therapy resistance, VEGF/VEGFR signalling, tivozanib, EGFR-directed therapies

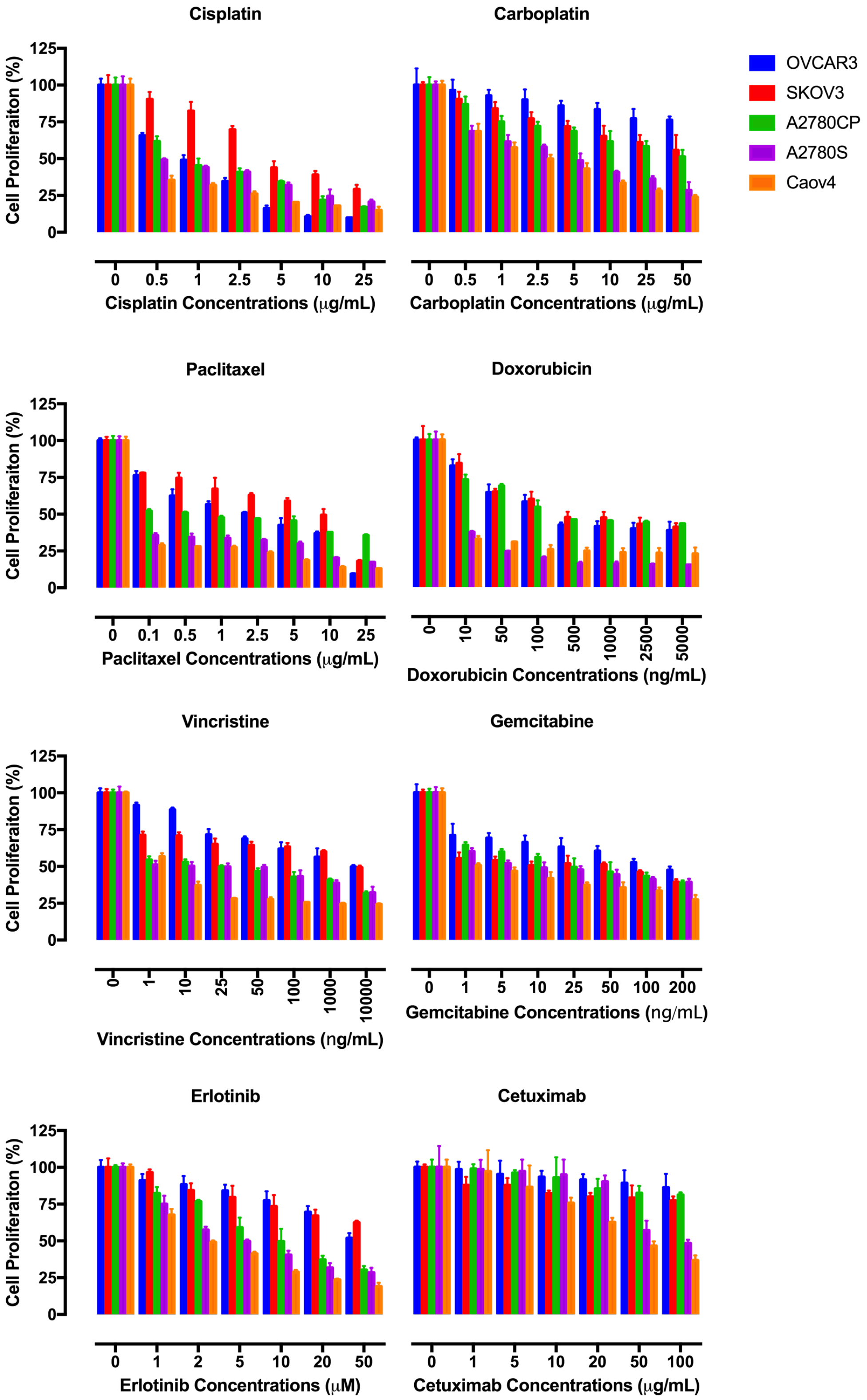

Supplementary Fig. 1

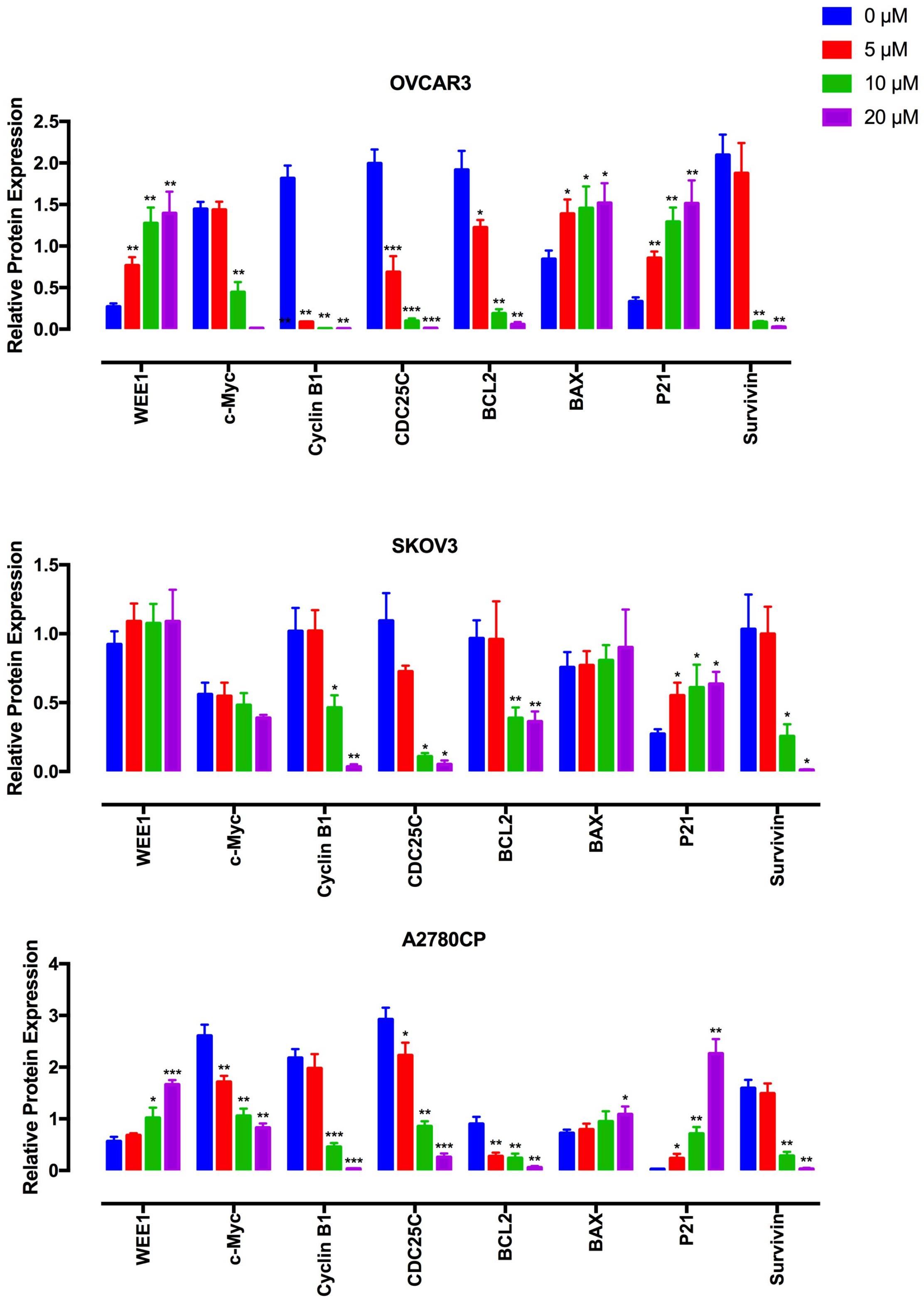

Supplementary Fig. 2

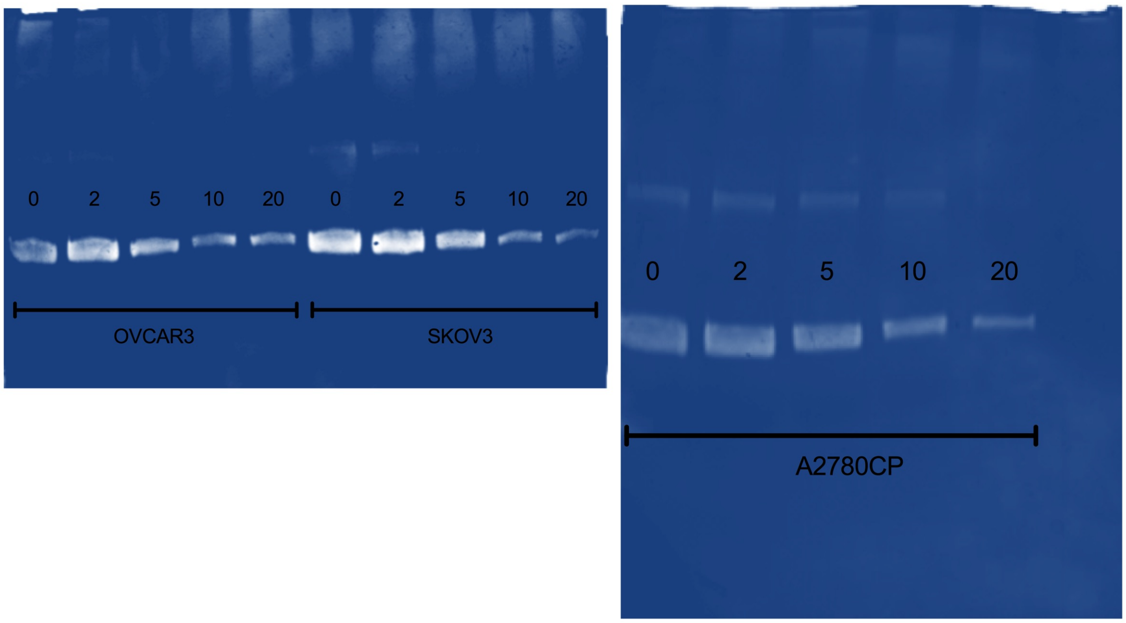

Supplementary Fig. 3

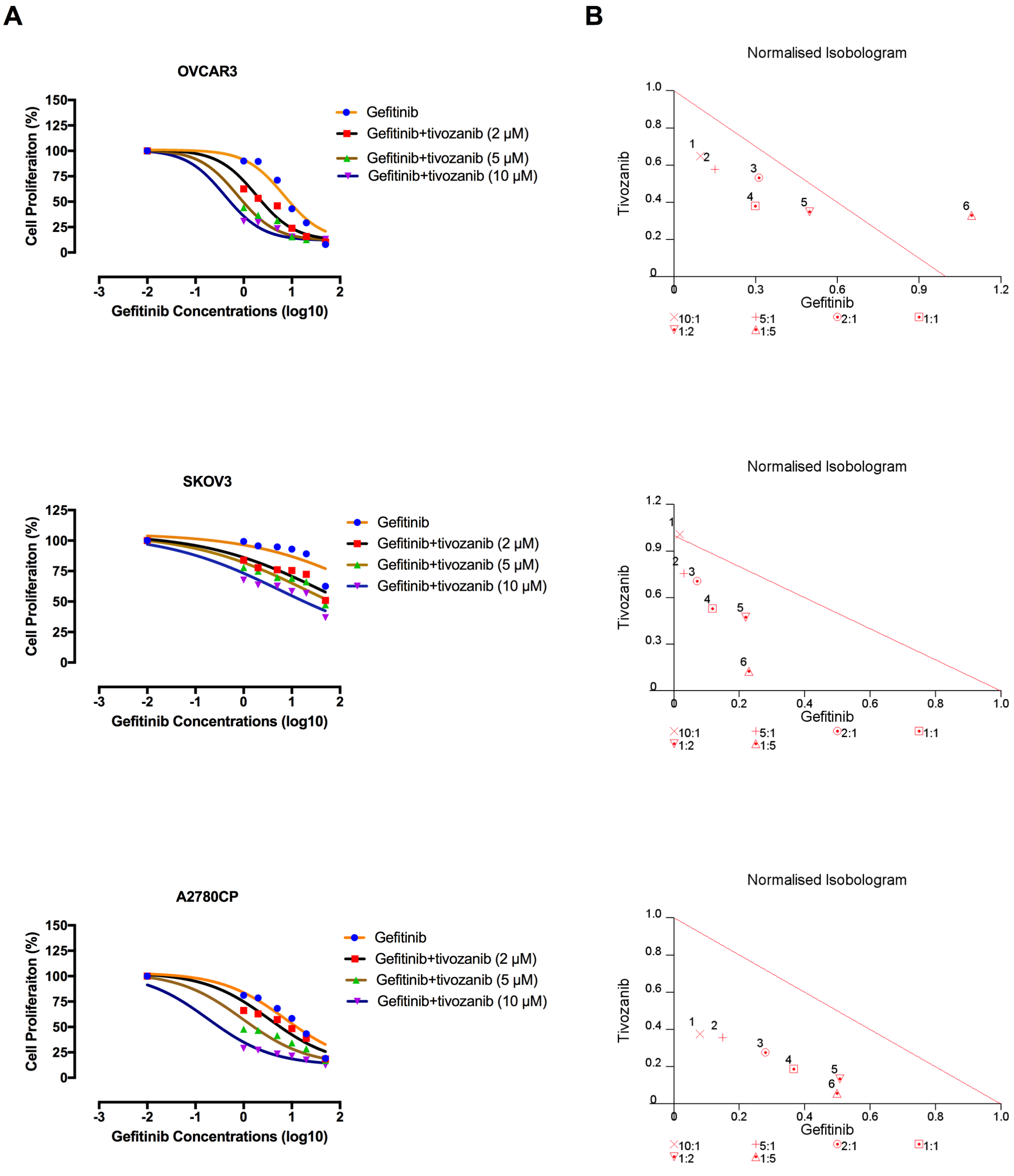

Supplementary Fig. 4

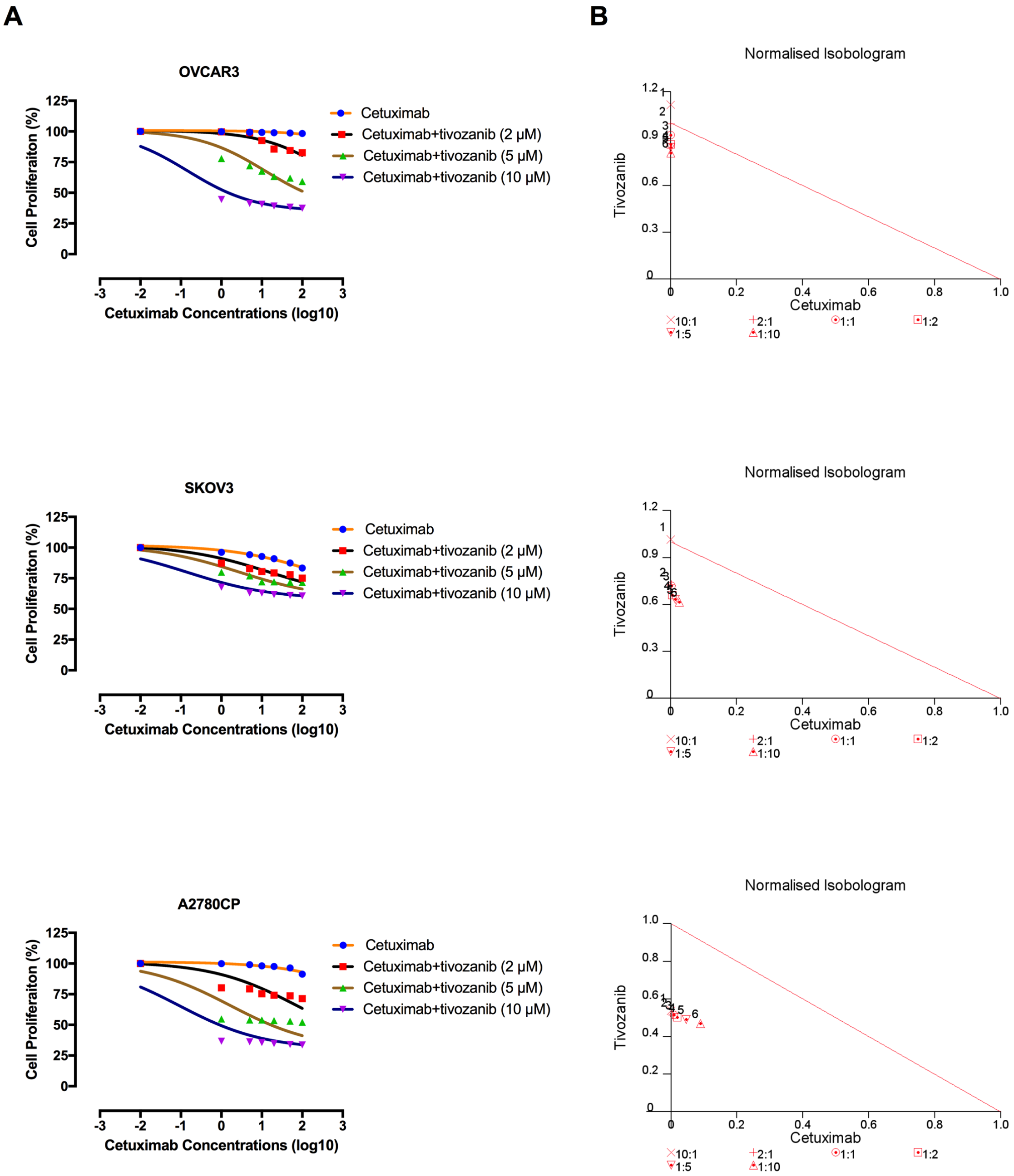

Supplementary Fig. 5

Supplementary Table 1. Combination index (CI) and dose reduction index (DRI) of apatinib and cispatin combination in OVCAR3, SKOV3 and A2780CP cells. DRI represents the order of magnitude of dose reduction that is allowed in combination for a given degree of effect as compared with the dose of each drug alone. ‘‘fa’’ denotes fraction affected.

**Concentrations**		**fa**	**CI**	**DRI**	
**Apatinib (µM)**	Cisplatin (µg/mL)			Apatinib	Cisplatin
		**OVCAR3**			
**10**	0.1	0.78	0.54	1.9	71.5
**10**	0.5	0.8	0.51	2.2	16.5
**10**	1	0.82	0.50	2.6	9.4
**10**	2.5	0.85	0.51	3.3	4.8
**10**	5	0.86	0.63	3.8	2.7
**10**	10	0.87	0.9	4.2	1.5
		**SKOV3**			
**10**	0.1	0.49	0.79	1.3	32
**10**	0.5	0.56	0.78	1.5	8.7
**10**	1	0.63	0.75	1.7	6
**10**	2.5	0.78	0.62	2.3	5.2
**10**	5	0.8	0.74	2.4	3
**10**	10	0.82	0.98	2.5	1.7
		**A2780CP**			
**10**	0.1	0.65	0.75	1.3	104.9
**10**	0.5	0.66	0.77	1.4	21.6
**10**	1	0.7	0.7	1.6	12.7
**10**	2.5	0.73	0.71	1.8	5.9
**10**	5	0.76	0.79	2.1	3.3
**10**	10	0.8	0.85	2.6	2.1

Supplementary Table 2. Nucleotide sequences of the primers used for qRT-PCR

Gene	Accession	Forward Primer	Reverse Primer	Amplicon
HPRT1	NM_000194	TGGACAGGACTGAACGTCTTG	CCAGCAGGTCAGCAAAGAATTTA	111
CDKN1A	NM_000389	CCTGTCACTGTCTTGTACCCT	GCGTTTGGAGTGGTAGAAATCT	130
c-MYC	NM_002467	GTCAAGAGGCGAACACACAAC	TTGGACGGACAGGATGTATGC	162
BIRC5	NM_001168	CCAGATGACGACCCCATAGAG	TTGTTGGTTTCCTTTGCAATTTT	152
IL6	NM_000600	ACTCACCTCTTCAGAACGAATTG	CCATCTTTGGAAGGTTCAGGTTG	149
IL8	NM_000584	GCTCTGTGTGAAGGTGCAGTT	ACCCAGTTTTCCTTGGGGTC	203
CCNB1	NM_031966	AATAAGGCGAAGATCAACATGGC	TTTGTTACCAATGTCCCCAAGAG	111
CDK1	NM_001786	AAACTACAGGTCAAGTGGTAGCC	TCCTGCATAAGCACATCCTGA	148
CDK2	NM_001798	CCAGGAGTTACTTCTATGCCTGA	TTCATCCAGGGGAGGTACAAC	90
CCNA2	NM_001237	TGGAAAGCAAACAGTAAACAGCC	GGGCATCTTCACGCTCTATTT	109
WEE1	NM_003390	AGGGAATTTGATGTGCGACAG	CTTCAAGCTCATAATCACTGGCT	160
GADD45A	NM_001924	GAGAGCAGAAGACCGAAAGGA	CACAACACCACGTTATCGGG	145
CHEK1	NM_001114122	ATATGAAGCGTGCCGTAGACT	TGCCTATGTCTGGCTCTATTCTG	183
CHEK2	NM_007194	TCTCGGGAGTCGGATGTTGAG	CCTGAGTGGACACTGTCTCTAA	205
CDC25B	NM_021873	GGCTGAGGAACCTAAAGCCC	CTTTCCGTCTACTGTCTGTAGGA	139
CDC25C	NM_001790	TCTACGGAACTCTTCTCATCCAC	TCCAGGAGCAGGTTTAACATTTT	98
SFN	NM_006142	TGACGACAAGAAGCGCATCAT	GTAGTGGAAGACGGAAAAGTTCA	133
MYT1	NM_004535	CGCCTCTGTTTCGGATGAATC	TGAATCTCGTCCTGTCCTGAC	75
ICAM1	NM_000201	AGCTTCGTGTCCTGTATGGC	TTTTCTGGCCACGTCCAGTT	70
VEGFA	NM_001025366	AGGGCAGAATCATCACGAAGT	AGGGTCTCGATTGGATGGCA	75
VEGFB	NM_003377	GAGATGTCCCTGGAAGAACACA	GAGTGGGATGGGTGATGTCAG	172
VEGFC	NM_005429	GAGGAGCAGTTACGGTCTGTG	TCCTTTCCTTAGCTGACACTTGT	96
VEGFD	NM_004469	ATGGACCAGTGAAGCGATCAT	GTTCCTCCAAACTAGAAGCAGC	81
VEGFR1	NM_002019	TTTGCCTGAAATGGTGAGTAAGG	TGGTTTGCTTGAGCTGTGTTC	117
VEGFR2	NM_002253	GGCCCAATAATCAGAGTGGCA	CCAGTGTCATTTCCGATCACTTT	109
VEGFR3	NM_182925	CTGGACCGAGTTTGTGGAGG	GTCACATAGAAGTAGATGAGCCG	138
BCL2	NM_000633	CAGGATAACGGAGGCTGGGATG	TTCACTTGTGGCCCAGATAGG	154

Supplementary Table 3

Concentrations (µM)		fa	CI	DRI	
**Tivozanib**	Gefitinib			Tivozanib	Gefitinib
			**OVCAR3**		
**10**	1	0.56	0.75	1.5	10.3
**10**	2	0.63	0.73	1.7	6.6
**10**	5	0.69	0.84	1.9	3.2
**10**	10	0.85	0.68	2.6	3.3
**10**	20	0.87	0.85	2.8	2
**10**	50	0.89	1.42	3	0.9
			**SKOV3**		
**10**	1	0.32	1.03	1	55.5
**10**	2	0.36	0.79	1.3	33.5
**10**	5	0.37	0.78	1.4	14
**10**	10	0.42	0.65	1.9	8.5
**10**	20	0.43	0.69	2.1	4.5
**10**	50	0.63	0.36	7.9	4.4
			**A2780CP**		
**10**	1	0.52	0.46	2.6	12.6
**10**	2	0.53	0.51	2.8	6.7
**10**	5	0.58	0.56	3.6	3.6
**10**	10	0.66	0.56	5.3	2.7
**10**	20	0.71	0.64	7.4	1.9
**10**	50	0.83	0.56	17.3	2

Supplementary Table 4

Concentrations		fa	CI	DRI	
**Tivozanib (μM)**	Cetuximab (μg/mL)			Tivozanib	Cetuximab
			**OVCAR3**		
**10**	1	0.22	1.12	0.9	3.47e+004
**10**	2	0.28	1	1	1.41e+004
**10**	5	0.32	0.92	1.1	1.1e+004
**10**	10	0.37	0.86	1.2	8416
**10**	20	0.38	0.84	1.2	3824
**10**	50	0.41	0.81	1.2	2478
			**SKOV3**		
**10**	1	0.32	1.02	1	1562
**10**	2	0.37	0.73	1.4	564
**10**	5	0.37	0.72	1.4	287.5
**10**	10	0.38	0.67	1.5	167.3
**10**	20	0.39	0.65	1.6	72.1
**10**	50	0.39	0.64	1.6	37.8
			**A2780CP**		
**10**	1	0.45	0.54	1.9	983.2
**10**	2	0.46	0.52	1.9	204.2
**10**	5	0.46	0.52	1.9	102.3
**10**	10	0.46	0.52	2	52.4
**10**	20	0.47	0.54	2	21.3
**10**	50	0.48	0.56	2.1	11.1

Supplementary legends

Supplementary Fig. 1: Chemosensitivity of the EOC cell lines to various drugs was determined by MTT assay and expressed as percentage of the vehicle-treated cells. The data represent mean ± SD of three independent experiments, each performed in triplicate.

Supplementary Fig. 2: Quantification of protein expression. Quantification of protein band intensities was done using the ImageJ software after normalising to the corresponding β-actin levels. Data are given as mean ± SD. Statistically significant values of $*p < 0.05$, $**p < 0.01$, and $***p < 0.001$ were determined compared with the control.

Supplementary Fig. 3: The effects of tivozanib on MMP-2 enzymatic levels. Gelatinolytic activities are visualized as clear bands against the blue background of stained gelatin. The zymograms are representative of three independent experiments with similar results.

Supplementary Fig. 4: (A) The effect of the time-sequenced tivozanib-gefitinib therapy on cell proliferation was investigated by MTT assay and shown by IC_{50} shift analysis. **(B)** Normalised isobolograms of combination of tivozanib (10 μM) and gefitinib (1, 2, 5, 10, 20 and 50 μM). The data were analysed using the CalcuSyn software. The diagonal line represents additivity. Data points located below the line indicate a synergistic drug-drug interaction and data points above the line indicate an antagonistic drug-drug interaction. The numbers under the isobolograms indicate the doses of tivozanib and gefitinib in combination.

Supplementary Fig. 5: (A) The effect of the time-sequenced tivozanib-cetuximab therapy on cell proliferation was investigated by MTT assay and shown by IC_{50} shift analysis. **(B)** Normalised isobolograms of combination of tivozanib (10 μM) and

cetuximab (1, 5, 10, 20, 50 and 100 μg/mL). The numbers under the isobolograms indicate the doses of tivozanib and cetuximab in combination.
